# The Impact of Pediatric Neutering in Dogs and Cats—A Retrospective Study

**DOI:** 10.3390/ani13152487

**Published:** 2023-08-01

**Authors:** Mariana Oliveira-Martins, Mariana Portugal, Luís Cardoso, Ana Martins-Bessa

**Affiliations:** 1 School of Agrarian and Veterinary Sciences, University of Trás-os-Montes e Alto Douro (UTAD), 5000-801 Vila Real, Portugal; mariana.limamartins99@gmail.com; 2 Coimbra Municipal Animal Shelter, Coimbra City Council, 3000-611 Coimbra, Portugal; marianap@utad.pt; 3 Department of Veterinary Sciences, UTAD, 5000-801 Vila Real, Portugal; lcardoso@utad.pt; 4 Animal and Veterinary Research Centre (CECAV), UTAD, 5000-801 Vila Real, Portugal; 5 Associate Laboratory for Animal and Veterinary Sciences (AL4AnimalS), UTAD, 5000-801 Vila Real, Portugal

**Keywords:** obesity, pediatric neutering, shelter medicine, undesirable behaviors, urinary incontinence

## Abstract

**Simple Summary:**

The impact of pediatric neutering has been a topic explored by various authors for several years; however, the existing literature reveals different, and sometimes even contradictory, results regarding the long-term effects of pediatric neutering. This study aimed to evaluate the effect of sterilization performed at a pediatric age on the occurrence of different conditions such as obesity, joint and urinary diseases and behavioral changes. This study has concluded that, in the study sample, there was not a statistically significant difference between the group neutered before 4 months of age and the group neutered at or after 6 months of age.

**Abstract:**

Surgical sterilization is a common procedure in veterinary practice; yet, the age at which to perform said procedure is still a controversial topic since the common practice of performing this surgery at the “conventional” age of 6 months is not supported by concrete scientific data. Therefore, it leaves space for veterinary professionals to opt for pediatric neutering, especially in the context of shelter medicine, since it allows the adoption of younger animals and is an important tool used to combat the overpopulation of stray animals by preventing their reproduction, even though some of the studies regarding the long-term effects of this approach seem to have contradictory results. Consequently, the present study aims to evaluate the impact of pediatric neutering on the occurrence of obesity, behavioral changes and urinary and joint diseases by posing an inquiry by means of telephone questionnaire to 105 owners of cats and dogs neutered and adopted from Coimbra Municipal Animal Shelter. The analysis of the gathered data did not show any statistically significant relationship between age of neutering and the presence of any of the aforementioned conditions in the animals under study.

## 1. Introduction

Traditionally, if there is no reproductive interest for the animal in question, cats and dogs are neutered from 6 months of age. On the other hand, pediatric neutering is defined as the ovariectomy/ovariohysterectomy or castration of a dog or cat during the first 6 to 16 weeks of age [1,2]. This latter approach has been heavily used in shelter medicine in order to allow the adoption of young, neutered puppies and kittens thus also reducing the number of animals that are abandoned or surrendered to animal shelters due to undesirable hormonally influenced behaviors [3]. In addition, this procedure is also beneficial in combating pet overpopulation, not only by inhibiting the ability of the adopted animals to procreate but also by preventing the reproduction of stray animals when performed within the context of spay and neuter programs [4].

Surgical sterilization is indeed one of the most common procedures performed in veterinary practices all over the world, and the most common reasons that motivate owners to seek this procedure for their pet are to function as a reliable contraception method and to reduce the risk of future health problems, since it is reported that this procedure has a positive effect on the prevention of reproductive tract diseases such as mammary neoplasia and pyometra in the female and benign prostatic hyperplasia and testicular cancer in the male [5,6]. Nevertheless, these procedures are also associated with some malign effects on the health of animals, especially dogs, which include an increased risk of developing prostate cancer and diabetes mellitus, a higher incidence of cranial cruciate ligament rupture and obesity, as well as an increased risk of developing tumors such as hemangiosarcoma, lymphoma and transitional cell carcinoma of the bladder, both in females and males [7,8]. It is, however, necessary to highlight that many health conditions, such as different types of neoplasia, have distinct occurrences depending on the species and even the breed in question. For this reason, it is important to take into consideration what breeds are used in the studies consulted and interpret the results with caution in order to avoid wrongly extrapolating information as truthful for the species as a whole when, in reality, the data only accurately represent a particular breed [5].

Many broad spectrum studies have been performed to further the research on this topic, including the one conducted by Hart et al. [9] and both of the studies performed by Spain et al. [10,11] pertaining to both the canine and feline species. However, some of the stated findings about the impact of neutering on the long-term health and well-being of animals seem to be contradictory in the different literary sources [9,10,11]. Specifically, in the case of female dogs, the optimum age of when to neuter is a contentious matter amongst veterinary professionals [12], and delaying the procedure until after the first heat is still an alternative used by many veterinarians, even with the lack of scientific data supporting this as a preferable approach [13]. Furthermore, the situation is even more complex regarding bitches that are available for adoption in animal shelters since pediatric neutering is essential to eliminate the risk of unwanted litters [14].

On the subject of the feline species, the study conducted by Howe et al. [5] advocates for pediatric neutering in this species, since they take into consideration the findings of previous studies in regards to the procedure’s impact on specific topics such as behavior [15] and orthopedic disorders like physeal fractures [16]. It concludes that the potential outcome of performing surgical sterilization is outweighed by the benefits it provides to cats if the owner is aware of the importance of maintaining an adequate weight in order to avoid the incidence of weight-related endocrine diseases. Some of the advantages of performing gonadectomy at 6 to 8 weeks in female cats include the substantial reduction in the risk of developing mammary neoplasia, while in male cats, the main benefit of doing this procedure at a pediatric age resides in the improvement of their behavior by decreasing aggression and sexual behaviors, including urine spraying, which is considered undesirable by many [5]. However, as with any procedure, it is not completely void of downsides, and studies like those of Spain et al. [11] and Moons et al. [17] have shown that, even though pediatric neutering reduces hyperactivity in cats, it also contributes to increased fearfulness in both males and females. 

Consequently, the decision about the age at which it is considered ideal to perform a neutering procedure in cats and dogs is still a controversial topic [18], not only due to the lack of fundamental scientific data that support the current age recommendation for neutering pets but also because a lot of veterinarians even now feel reluctant to perform the surgery at an early age since the long-term effects are not completely known [19]. With that in mind, this retrospective study aimed to evaluate the impact that pediatric neutering has on the health and well-being of cats and dogs in the short-term by comparing the effects that ovariohysterectomy or ovariectomy and orchiectomy procedures have in a population of animals less than 4 months of age with the effects that the same procedures have when performed in a population of animals of the same species neutered at or after 6 months of age.

## 2. Materials and Methods

### 2.1. Study Method

The survey method chosen to collect information for this study was a telephone questionnaire. This option was selected because this type of consultation allows for a more flexible approach in which the owners can freely respond to the questions posed and, when necessary, they can be asked to expand on certain topics in order to obtain reliable and valid information, which would not have been possible when sending a printed version of the questionnaire by postal mail. Additionally, this methodology made it possible to reach many more people in a shorter period of time when compared to, for example, carrying out this survey in person on a date to be arranged with each owner.

The most relevant disadvantage of the selected approach in contrast with the latter one presented is the inability to visually assess the animal in question; however, to try to solve this issue, the questionnaire was designed with several questions on the same topic to ensure that the resulting answer represented reality as close as possible.

### 2.2. Ethical Approval

This study received the approval of the person in charge of data protection at the Coimbra City Council who allowed the use of the internal database in order to contact each owner by telephone. 

At the beginning of each call, the approached owner was asked if they consented to answering the questionnaire after being informed that the data collected could be used for scientific purposes, always assuring the owner’s anonymity. Additionally, all the data used in this retrospective study were exclusively obtained through a verbal conversation by telephone with the owner of the animal. Thus, there was no possibility of inflicting any harm to the animal since no procedures were physically performed. 

The present study was retrospective in nature, which means that the surgical interventions were performed to neuter the animals and were in no way influenced by the conduction of this study. Consequently, carrying out this study was completely innocuous to the animals of interest, and the publication of the data obtained had no risk associated with the animals or people involved.

### 2.3. Target Population

To be eligible to participate in this study, the animals should had been neutered and adopted from Coimbra Municipal Shelter between January 2016 and December 2020.

Alongside this, cats that were neutered during this time but were released in the sequence of a trap–neuter–release (TNR) program were excluded from this study. Animals that appeared as “dead” or “missing” on the national database were also excluded from this study.

After applying the inclusion and exclusion criteria, the target population was identified, and it was composed of 837 individuals. From this, the animals were sorted into two groups: those neutered before 4 months of age (542 animals), which were considered the “pediatric” group, and those that were neutered after 6 months of age (295 animals), which were considered the “adult” group. In the target population, none of the animals underwent surgical intervention between 4 and 6 months of age; hence, the absence of such a category of animals.

### 2.4. Study Sample Characterization

The study sample, representing approximately 30% of the target population, was selected by means of simple random sampling. This method assured an absence of biased results, since all the animals that comprised the target population had the same chance of being selected to be included in the study sample.

**Figure 1:**
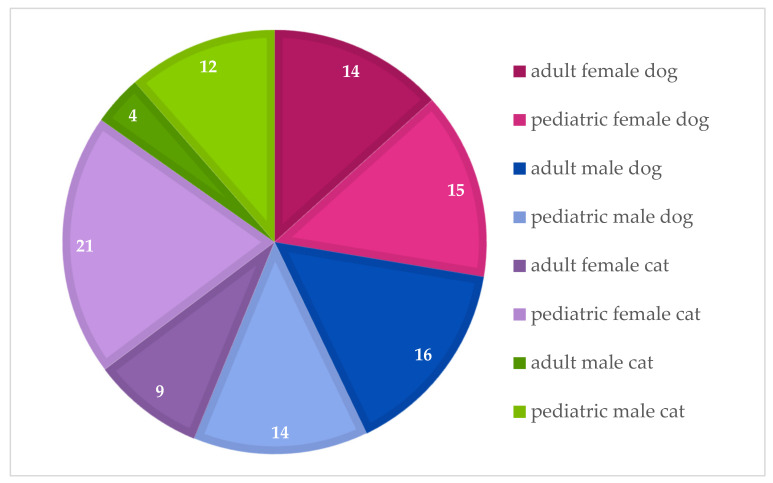
Characterization of the participants in the study by group, regarding the animals’ species, gender and age at neutering (before 4 months of age and at or after 6 months of age).

Then, a questionnaire was performed by means of telephone questionnaire in order to collect the information of interest, and, within the sample group, 105 owners agreed to answer the questionnaire regarding their pet in full (Figure 1).

The ages of the animals at the time of the telephone survey varied between 2 years and 2 months and 13 years and 11 months, and the average age of the animals that constituted the sample was 5 years and 1 month.

### 2.5. Experimental Design

The telephone survey was carried out from the 9 November to the 29 December 2022. This questionnaire (Appendix A) consisted of 23 questions, 8 of which were aimed at describing the animal, so they were answered based on the information that was in the Municipal Shelter’s database. The remaining 15 questions were aimed at studying the occurrence of four conditions: obesity; diseases pertaining to the urinary system, such as urinary incontinence, infection and obstruction of the lower urinary tract; behavioral problems, including reactions related to aggression, fear and urine marking; and lastly, joint diseases.

These main variables under study were selected after consulting some of the published sources on the topic and crossing that information with the current average age of the study sample. In other words, as the animals were selected from January 2016 and beyond, which means that many of the animals would still be young adults, it was expected that most of the study sample would not yet be affected by some types of tumors, and, consequently, neoplasia was not a variable of great interest in this study.

Finally, this study was reliant on each of the owners’ detailed responses in order to allow the correct identification of the animals affected by one or more of the abovementioned diseases or behavioral problems. Therefore, to ensure an objective classification of all the animals and to correctly assign them to their respective groups, the answers given by the owner had to be compatible with a predetermined set of descriptive phrases (Appendix B) for the animal to be considered affected by a certain condition.

### 2.6. Statistical Analysis

The data obtained were analyzed statistically, and the differences among groups were assessed using Pearson’s chi-squared test or, alternatively, Fisher’s exact test. The adopted confidence interval (CI) was 95%, and the differences were considered to be significant at *p* < 0.05. 

Independent variables with significant difference between categories (*p* < 0.05) were selected for logistic regression analysis to identify independent risk factors by calculating odds rations (OR) and their 95% CI [20]. All the statistical tests were performed using the IBM SPSS 28 statistics package^®^.

## 3. Results

Of the 105 animals, 47 were cats and 58 were dogs. A total of 62 were neutered before the first 4 months of age (“pediatric” group), and the remaining 43 were neutered at or after 6 months of age (“adult” group) (Table 1).

After all the 105 questionnaires were fully answered, the collected data were used to evaluate if each animal presented one or more of the conditions under study (Table 2). None of the owners stated that their animal had any kind of joint disease, and the only cases of orthopedic problems were associated with traumatic events that culminated in fractures of long bones, so, logically, this variable was not taken into consideration in the data analysis. 

Both species were evaluated separately, assuming as independent variables the gender, the age at neutering and, for the dog population, the weight of the animal at the time of the telephone survey.

### 3.1. Dogs

(a) Gender

In the dog population, regarding gender (Table 3), the only statistically significant difference found was between the presence of urinary diseases and the female gender (*p* = 0.021), and after analyzing the occurrence of each urinary disease under study, a relationship between being a female dog and having a history of lower urinary tract infection, more specifically bacterial cystitis, was also found (*p* = 0.048). Following this, a logistic regression analysis was performed in order to further explore this topic, but we were not able to identify a statistically significant risk factor.

(b) Weight

Regarding the weight categories of dogs (small, medium or large), a relationship between weight and the presence of a body condition score (BCS) >6 was found (*p* = 0.015) (Table 4). By exploring this topic further (Table 5) and comparing the three categories, it was established that the statistically significant difference lied between the small and medium weight categories (*p* = 0.010), and after applying logistic regression, it was confirmed that the medium weight category was indeed the risk factor associated with a higher occurrence of obesity (OR = 14.57; 95% CI: 1.68–126.24) (Table 6). However, as obesity is a multifactorial and complex issue, the present study was unable to determine if other factors contributed to the elevated BCS observed.

(c) Age at neutering

Regarding the age of the animal at the moment of the neutering procedure, the present study did not find any statistically significant difference between either one of the groups (<4 months and ≥6 months) and the presence of any of the conditions under study (Table 7).

(d) Time elapsed between surgery and survey

Since the data used in this study only included animals adopted between 2016 and 2020, it was important to assess whether the time elapsed between the date of surgery and the date of the survey influenced the occurrence of certain conditions in the animals that constituted the sample. While testing the hypothesis that the elapsed time between surgery and the survey conditions the presence of these diseases, or, in other words, if the animals intervened at an older age (5 to 7 years) have a higher occurrence of the pathological conditions under study compared to the animals more recently neutered (2 to 4 years), the present study did not find any statistically significant difference between either one of the groups and the presence of any of the conditions under study (Table 8).

### 3.2. Cats

The present study did not find any statistically significant difference between either of the independent variables and the occurrence of the conditions under study (Table 9, Table 10 and Table 11). 

## 4. Discussion

To allow the statistical analysis of the varied information gathered from the inquiries, the data were simplified into categories. Regarding the weight of the animal, the dogs included in this study were sorted into three categories of weight: small (≤10 kg), medium (>10 kg and ≤25 kg) and large (>25 kg). Additionally, since the format of this study did not allow for a physical exam of the animal performed by a medical expert, the body score evaluation of the animal relied on the verbal description given by the owner, so, in an attempt to receive an accurate assessment, the questionnaire included several questions regarding the topic, including describing if the owner felt the waist and ribs of the animal and if the pet had suffered a considerable weight gain recently, and the answers obtained were appraised together so that it was possible to determine if the animal was overweight or not.

The results pertaining to the variables undesirable behaviors and urinary tract diseases were also divided into categories, being sorted into four (fear, aggression, urine marking and roaming) and three (urinary incontinence, urinary obstruction or presence of urinary calculi, and lower urinary tract infection) groups, respectively. After the required adjustment of the gathered data, the analysis did not show a statistically significant difference between the group that was neutered after 6 months of age and the group that was neutered before 4 months of age regarding the occurrence of the different conditions under study in both species. This conclusion is compatible with some of the previous studies that also focused on the evaluation of these variables [5,10,11].

Despite these findings, in regard to urinary incontinence, studies like Spain et al. [10] defend the existence of a relationship between pediatric neutering and the occurrence of urinary incontinence in bitches. Additionally, studies like Holt et al. [21] and more recently de Bleser et al. [22] add that there seems to exist a relationship between a dog being of a large weight category and the incidence of this disease. However, as stated above, the results of the present study do not support the previous claims, as the results obtained were more similar to the conclusions of the study conducted by Forsee et al. [23], where there was not a statistically significant relationship between neutering female dogs before 24 weeks of age and the occurrence of urinary incontinence. 

Despite this, some statistically significant relationships were still found, such as the correlation between a dog being of a medium weight category and being obese (*p* = 0.015), but this finding might not be indicative of a true cause-and-effect relationship. For example, in this particular case, it is most likely that the relationship between being a medium dog and being obese was due to factors that were not explored in this study, such as the animal’s meal regimen or daily level of physical activity, which can be more or less conducive to difficulties in controlling weight gain. This should be taken into consideration in further studies since the information collected in the questionnaire regarding things such as the number of meals given per day or the living regimen of the animal was not taken into consideration in the statistical analysis of this present study.

It is necessary to highlight that this study had some limitations that were not just inherent to the fact that it was a retrospective study, meaning it was reliant on analog records that were not all documented by the same person. But also, the intra- and post-surgical mortality levels were not taken into consideration since that information was not available in the internal database of the shelter. Additionally, even though our resource of potential individuals to inquire about the occurrence of the conditions under study was made up of 837 animals, the sample only comprised 105 animals whose owners consented to being included in the present study. This was due to the time constraints in developing this study as well as the fact that some of the owners’ contact information that was initially provided to the shelter was outdated, which made it impossible to perform the telephone survey. Consequently, the relatively small sample size may have influenced the results of the present study and may also account for a larger margin of error.

The average age of the sample was only 5 years and 1 month, which means the animals in this study might still not have developed some of the diseases of interest in this study. Additionally, even though the present study did not find any statistically significant difference regarding the time elapsed between the pet’s surgery and the date of the questionnaire, in both species, the longest time recorded was only 6 years and 7 months, which might not have been sufficient time to allow an accurate evaluation of the long-term effects that pediatric neutering might have on the health and well-being of these animals since this follow-up period was relatively short. And lastly, since the animals included in this study were all adopted from Coimbra Municipal Shelter, the data associated with the sample might not accurately describe the national reality since most of the animals stayed in the nearby geographical area.

## 5. Conclusions

The impact of pediatric neutering has been a topic explored by several authors for many years; however, the existing literature reveals different, and sometimes even contradictory, results regarding the effects of this procedure. The present study aimed to explore this topic, and the data obtained suggest that sterilization performed before 4 months of age and at or after 6 months of age has a similar impact on the health and well-being of the animal that underwent surgery, both in the canine and feline species. Nevertheless, given the aforementioned limitations of this study, especially those regarding the small sample size, the relatively short follow-up period of the animals under study, and the disregard of the mortality associated with the procedures, it is important to explore this topic in more detail by supporting studies that compare large groups of individuals neutered at pediatric age (before 4 months old) and neutered at a “conventional” age (6 months old). In addition, it is also important to include a third group of intact animals in the comparison, if possible, assuring the evaluation of all those animals for a long time, preferably until their death. These types of studies would allow the gathering of concrete scientific data that support the safety for the long-term health of animals subjected to pediatric neutering, allowing veterinary professionals to feel confident in using pediatric neutering as a method for sterilization when necessary.

## Figures and Tables

**Table 1:** Characterization of the sample.

Independent Variable	Category	Count	Percentage
Species	Feline	47	44.76%
	Canine	58	55.24%
Gender	Female	59	56.19%
	Male	46	43.81%
Weight at the time of the survey (dogs only)	Small (≤10 kg)	18	31.03%
	Medium (10–25 kg)	26	44.83%
	Large (>25 kg)	14	24.14%
Age of neutering	<4 months	62	59.05%
	≥6 months	43	40.95%
Time between surgery and survey	2 to 4 years 5 to 7 years	55 50	52.38% 47.62%

**Table 2:** Numerical count of the animals affected by the conditions under study in each group.

Condition	AFD	PFD	AMD	PMD	AFC	PFC	AMC	PMC	Total
Obesity	5	1	7	5	4	6	2	5	35
Undesirable behaviors	9	9	10	11	3	11	3	4	60
Fear reaction	5	7	4	4	2	8	2	4	36
Aggression	4	2	2	5	1	5	0	0	19
Urine marking	0	1	3	2	0	0	0	0	6
Roaming	3	3	4	0	0	1	1	1	13
Urinary tract diseases	2	3	0	0	0	2	0	1	8
Urinary obstruction	0	0	0	0	0	2	0	1	3
Lower urinary tract infection	2	2	0	0	0	1	0	0	5
Urinary incontinence	0	3	0	0	0	0	0	0	3
Joint diseases	0	0	0	0	0	0	0	0	0

AFD: adult female dog; PFD: pediatric female dog; AMD: adult male dog; PMD: pediatric male dog; AFC: adult female cat; PFC: pediatric female cat; AMC: adult male cat; PMC: pediatric male cat. (“adult” = neutered at or after 6 months; “pediatric” = neutered before 4 months).

**Table 3:** Relationship between the gender and the presence of the conditions under study in the canine population.

Gender	Frequency	Obesity	One or More Undesirable Behaviors	Fear Reaction	Aggression	Urine Marking	Roaming	Urinary Tract Diseases	Urinary Incontinence	Lower Urinary Tract Infection	Urinary Obstruction
Female	n	6	18	12	6	1	6	5	3	4	0
	%	21.4%	64.3%	42.9%	21.4%	3.6%	21.4%	17.9%	10.7%	14.3%	0.0%
Male	n	12	21	8	7	5	4	0	0	0	0
	%	40.0%	70.0%	26.7%	23.3%	16.7%	13.3%	0.0%	0.0%	0.0%	0.0%
*p* value		0.214	0.854	0.308	1.000	0.195	0.499	**0.021**	0.106	**0.048**	N/A

N/A—not applicable.

**Table 4:** Relationship between the weight category and the presence of the conditions under study in the canine population.

Weight Categories	Frequency	Obesity	One or More Undesirable Behaviors	Fear Reaction	Aggression	Urine Marking	Roaming	Urinary Tract Diseases	UrinaryIncontinen ce	Lower Urinary Tract Infection	Urinary Obstruction
Small (≤10 kg)	n	5	8	4	4	1	0	2	2	2	0
	%	35.7%	57.1%	28.6%	28.6%	7.1%	0.0%	14.3%	14.3%	14.3%	0.0%
Medium (10–25 kg)	n	12	19	11	5	1	5	2	0	2	0
	%	46.2%	73.1%	42.3%	19.2%	3.8%	19.2%	7.7%	0.0%	7.7%	0.0%
Large (>25 kg)	n	1	12	5	4	4	5	1	1	0	0
	%	5.6%	66.7%	27.8%	22.2%	22.2%	27.8%	5.6%	5.6%	0.0%	0.0%
*p* value		**0.015**	0.591	0.527	0.796	0.130	0.111	0.666	0.150	0.280	N/A

N/A—not applicable.

**Table 5:** Relationship between the medium and small dog weight categories and the occurrence of obesity.

			Yes	No	Total
Weight categories Medium × Small	Medium	Count (n)	12	14	26
		Percentage (%)	46.2%	53.8%	100%
	Small	Count (n)	1	17	18
		Percentage (%)	5.6%	94.4%	100%
Total		Count (n)	13	31	44
		Percentage (%)	29.5%	70.5%	100%
*p* value		*p* = **0.010**			

**Table 6:** Logistic regression analysis regarding the weight categories of the canine species and the occurrence of obesity.

Weight Categories	Coefficients	Standard Errors	Wald Test	Degrees of Freedom	*p* Value	Odds Ratio (OR)
Small weight category (reference category)			5.933	2	0.051	
Large weight category	2.245	1.170	3.680	1	0.055	9.444
Medium weight category	2.679	1.102	5.914	1	**0.015**	**14.571**
	−2.833	1.029	7.581	1	0.006	0.059

**Table 7:** Relationship between the age at neutering and the presence of the conditions under study in the canine population.

Age at Neutering	Frequency	Obesity	One or More Undesirable Behaviors	Fear Reaction	Aggression	Urine Marking	Roaming	Urinary Tract Diseases	Urinary Incontinence	Lower Urinary Tract Infection	Urinary Obstruction
Pediatric (<4 months)	n	6	20	11	7	3	3	3	3	2	0
	%	20.7%	69.0%	37.9%	24.1%	10.3%	10.3%	10.3%	10.3%	6.9%	0.0%
Adult (≥6 months)	n	12	19	9	6	3	7	2	0	2	0
	%	41.4%	65.5%	31.0%	20.7%	10.3%	24.1%	6.9%	0.0%	6.9%	0.0%
*p* value		0.156	1.000	0.782	1.000	1.000	0.297	1.000	0.237	1.000	N/A

N/A—not applicable.

**Table 8:** Relationship between time elapsed between the date of surgery and the date of the survey and the presence of the conditions under study in the canine population.

Time between Surgery and Survey	Frequency	Obesity	One or More Undesirable Behaviors	Fear Reaction	Aggression	Urine Marking	Roaming	Urinary Tract Diseases	Urinary Incontinence	Lower Urinary Tract Infection	Urinary Obstruction
2 to 4 years	n	13	21	8	7	4	5	1	0	1	0
	%	43.3%	70.0%	26.7%	23.3%	13.3%	16.7%	3.3%	0.0%	3.3%	0.0%
5 to 7 years	n	5	18	12	6	2	5	4	3	3	0
	%	17.9%	64.3%	42.9%	21.4%	7.1%	17.9%	14.3%	5.2%	10.7%	0.0%
*p* value		0.070	0.854	0.308	1.000	0.671	1.000	0.187	0.106	0.344	N/A

N/A—not applicable.

**Table 9:** Relationship between the gender and the presence of the conditions under study in the feline population.

Gender	Frequency	Obesity	One or More Undesirable Behaviors	Fear Reaction	Aggression	Urine Marking	Roaming	Urinary Tract Diseases	Urinary Incontinence	Lower Urinary Tract Infection	Urinary Obstruction
Female	n	11	13	9	5	0	1	3	0	1	3
	%	35.5%	41.9%	29.0%	16.1%	0.0%	3.2%	9.7%	0.0%	3.2%	9.7%
Male	n	6	8	7	1	0	2	0	0	0	0
	%	37.5%	50.0%	43.8%	6.3%	0.0%	12.5%	0.0%	0.0%	0.0%	0.0%
*p* value		1.000	0.828	0.494	0.648	N/A	0.264	0.541	N/A	1.000	0.541

N/A—not applicable.

**Table 10:** Relationship between the age at neutering and the presence of the conditions under study in the feline population.

Age at Neutering	Frequency	Obesity	One or More Undesirable Behaviors	Fear Reaction	Aggression	Urine Marking	Roaming	Urinary Tract Diseases	Urinary Incontinence	Lower Urinary Tract Infection	Urinary Obstruction
Pediatric (<4 months)	n	11	15	12	5	0	2	3	0	1	3
	%	33.3%	45.5%	36.4%	15.2%	0.0%	6.1%	9.1%	0.0%	3.0%	9.1%
Adult (≥6 months)	n	6	6	4	1	0	1	0	0	0	0
	%	42.9%	42.9%	28.6%	7.1%	0.0%	7.1%	0.0%	0.0%	0.0%	0.0%
*p* value		0.772	1.000	0.742	0.653	N/A	1.000	0.544	N/A	1.000	0.544

N/A—not applicable.

**Table 11:** Relationship between time elapsed between the date of surgery and the date of the survey and the presence of the conditions under study in the feline population.

Time between Surgery and Survey	Frequency	Obesity	One or More Undesirable Behaviors	Fear Reaction	Aggression	Urine Marking	Roaming	Urinary Tract Diseases	Urinary Incontinence	Lower Urinary Tract Infection	Urinary Obstruction
2 to 4 years	n	10	12	11	1	0	1	1	0	0	1
	%	40.0%	48.0%	44.0%	4.0%	0.0%	4.0%	4.0%	0.0%	0.0%	4.0%
5 to 7 years	n	7	9	5	5	0	2	2	0	1	2
	%	31.8%	40.9%	22.7%	22.7%	0.0%	9.1%	9.1%	0.0%	4.5%	9.1%
*p* value		0.781	0.846	0.220	0.085	N/A	0.593	0.593	N/A	0.468	0.593

N/A—not applicable.

## Data Availability

Raw data can be provided on request.

## Appendix A

Telephone Inquiry: (*answer categories*)

* = Information filled out according to the database

1. What is your animal’s name? * (*open*)
2. What species is your animal? * (*dog*/*cat*)
3. What is the animal’s sex? * (*female*/*male*)
4. What is your animal’s breed? * (*open*)
5. What is the animal’s age at the date of the questionnaire? * (*open*)
6. When was the date of surgery? * (*open*)
7. What was the animal’s age at the date of surgery? * (*open*)
8. What is the animal’s weight category (only applies to dogs)? * (*small*/*medium*/*large*)
9. What is the animal’s weight at the date of the questionnaire? (*open*)
10. How many meals do you feed your pet per day? (*1 meal per day*/*2 meals per day*/*ad libitum*)
11. What kind of food do you feed the animal? (*dry food*/*wet food*/*homemade food*/*of any type*)
12. Do you often provide your pet with treats (i.e., crunchy treats, rawhide, jerky, dental chews etc.)? (*yes*/*no*)
13. Are you able to feel you pet’s ribs when you touch it? (*yes*/*no*)
14. Are you able to see your pet’s waist behind its rib cage from above? (*yes*/*no*)
15. Has your pet experienced a considerable weight gain at any stage of its life? (*yes*/*no*)
16. Does your pet have access to the outside? Do you have a backyard, or do you take frequent walks with the animal? (*strictly indoor*/*strictly outdoor*/*mixed* (*access to both indoors and outdoors*)/*other arrangement*)
17. Has your animal, since it was adopted, ever shown any undesirable behavior, such as aggression, marking territory or objects with urine, among others? (*yes*/*no*)
  17.1. If so, how does your pet react? (aggressive behavior within its territory or guarding instinct/negative reaction to food handling/aggression towards other animals or other people/marking territory or objects with urine/fearful and nervous/separation anxiety or destructive behavior when left alone at home/escape from home/other)
18. Does your pet have urinary incontinence (i.e., involuntary urine leakage, while sleeping or when standing still)? (*yes*/*no*)
  18.1. If so, when did it start to occur? (*open*)
19. Have you ever noticed any urinary problems in your pet (i.e., dysuria, hematuria, etc.)? (*yes*/*no*)
20. Is there any other problem or illness that your pet has developed since it was adopted? (*dermatitis*/*vaginitis*/*cystitis*/*ovary remnant syndrome*/*diabetes mellitus*/*hypothyroidism*/*joint diseases*/*neoplasia* (*i.e., mammary tumors, osteosarcoma, etc.*)/*autoimmune diseases* (*i.e., pemphigus, lupus, etc.*)/*other*)
21. Has your pet had any additional surgery since it was adopted? (*yes*/*no*)
  21.1. If so, what and when was the surgery? (*open*)
22. Has your pet been annually monitored by a veterinarian? (*yes*/*no*)
23. Does your pet have all recommended vaccinations and deworming up to date? (*yes*/*no*)

## Appendix B

**Table A1:** Model descriptive phrases for each disease and behavioral problem under study.

Conditions	Canine	Feline
Obesity	The owner described that the ribs were not easily palpable, since there was some excess fat, they were not palpable without significant pressure, or they weren’t palpable at all. The owner described the absence of an identifiable waist viewed from above. The animal is being followed by their veterinarian in order to lose weight, and the owner was able to relay the body condition score given by the veterinarian.	
Aggression	The animal has shown territorial aggression or resource guarding by growling or snarling at another animal and/or person. The animal has gravely bitten or growled at someone when handling their food. The animal has gravely bitten another animal and/or person. The animal lunges at other animals and/or people in an attempt to attack them. The owner has sought help from a certified trainer or behaviorist specialist to address this type of behavior.	The animal has shown territorial aggression or resource guarding by growling or hissing at another animal and/or person. The animal has gravely bitten or scratched another animal and/or person. The animal lunges at other animals and/or people in an attempt to attack them. The owner has sought help from a certified trainer or behaviorist specialist to address this type of behavior. The owner has been advised to use “calming” sprays or diffusers in order to manage the behavior.
Fear	The animal hides or avoids new people and/or animals. The animal hides or whimpers when confronted with overwhelming stimulus (i.e., loud noises form fireworks or thunderstorms). The owner has sought help from a certified trainer or behaviorist specialist to address this type of behavior. The animal has been prescribed medication in order to manage the behavior.	
Urine marking	The animal marks areas that are deemed unacceptable by the owner with urine (i.e., furniture, walls, plants, etc.).	The animal exhibits urine “spraying”, by releasing a small amount of urine against a surface.
Roaming	The owner described that the pet had left the property and could not find the animal for at least 24 h.	
Urinary incontinence	The owner described that the animal leaves “urine spots” on the place where it was lying or sleeping. Animal has been diagnosed by a veterinarian with urinary incontinence.	
Lower urinary tract infection	The owner sought treatment, and the animal was diagnosed by a veterinarian with a lower urinary tract infection.	
Urinary obstruction	The owner sought treatment, and the animal was diagnosed by a veterinarian with urinary obstruction or the presence of calculi in the urinary tract.	
